# Anatomical Variations in Bilateral Hand Oligodactyly: A Case Analysis of Cleft Hand

**DOI:** 10.3390/diagnostics16020275

**Published:** 2026-01-15

**Authors:** Mi-Sun Hur, Jae Woo Shim, Ju-Hyeon Bae, Jong-Sun Kang, Kwang Il Nam, Chang-Seok Oh

**Affiliations:** 1 Department of Anatomy, Daegu Catholic University School of Medicine, Daegu 42472, Republic of Korea; 2 Department of Orthopedic Surgery, Samsung Medical Center, Sungkyunkwan University School of Medicine, Seoul 06351, Republic of Korea; 3 Department of Molecular Cell Biology, Sungkyunkwan University School of Medicine, Suwon 16419, Republic of Korea; 4 Department of Anatomy, Chonnam National University Medical School, Gwangju 58128, Republic of Korea; 5 Department of Anatomy, Sungkyunkwan University School of Medicine, 2066, Seobu-Ro, Jangan-Gu, Suwon 6419, Republic of Korea

**Keywords:** congenital anomaly, deformity, forearm, hand, malformation, oligodactyly

## Abstract

**Background:** Hand oligodactyly is a rare congenital malformation characterized by fewer than five digits. Although several clinical and radiological studies have been reported, detailed anatomical investigations remain scarce. This study aimed to describe the morphological characteristics of bilateral hand oligodactyly through dissection. **Case Presentation:** A bilateral hand oligodactyly with thumb and 3 fingers on each hand was found in a 77-year-old Korean female donor. The study was approved by the Institutional Review Board of Sungkyunkwan University (IRB No. SKKU 2023-02-012). **Results:** Diverse variations were observed in most structures of the hand, including the palmar aponeurosis, tendons of the flexor digitorum superficialis, the flexor digitorum profundus, and the flexor pollicis longus, lumbricals, interossei, and metacarpals. Connections between flexor and extensor tendons were also found, and each variation differed between the two hands. **Conclusions:** These structural differences highlight the developmental complexity of hand oligodactyly and offer valuable insights for facilitating appropriate surgical strategies. This study has also provided new insights into congenital anomalies that underscore their relevance in developmental biology.

## 1. Introduction

Hand oligodactyly is a congenital anomaly in which some digits are absent, which is a common feature of the congenital malformations of the upper limb. The latter can be classified into four primary categories: radial dysplasia (also referred to as radial longitudinal deficiency), ulnar dysplasia (ulnar longitudinal deficiency), cleft hand (also known as split hand, ectrodactyly, lobster claw, or central oligodactyly), and symbrachydactyly [1,2,3].

Previous studies have documented various cases of hand oligodactyly and highlighted the improvements in hand function that are possible following surgical interventions [1,3,4,5,6,7,8,9,10,11,12,13,14,15,16]. For example, Baek and Kim (2016) [5] demonstrated that surgical techniques such as syndactyly division, corrective osteotomy, flexor plasty, and joint arthrolysis can enhance both the functionality and appearance of the hand by addressing the specific anatomical and functional needs of specific patients. Similarly, Sykes (1991) [13] described that both typical and atypical cleft-hand deformities vary in severity and so require individualized surgical planning. That author also mentioned that surgical treatments should be performed early, and that these procedures often involve complex reconstructions of both bone and soft tissue in severe cases.

Most of the previous studies have primarily focused on external contours, radiographic findings, and ultrasound appearance, with detailed anatomical investigations of internal structures in hand oligodactyly being rare. For instance, a British professional tennis player with congenital oligodactyly has achieved remarkable competitive success, bringing broader awareness to this anomaly. However, a thorough anatomical study of oligodactyly has yet to be reported.

This study aimed to elucidate the detailed anatomical variations in cases of bilateral hand oligodactyly. The analysis has provided insights into the structural characteristics and variations associated with this rare malformation. The results of this study are also expected to deepen the understanding of the developmental biology underlying congenital hand anomalies and provide a foundation for developing more effective surgical strategies for these deformities.

## 2. Case Presentation

A case of bilateral hand oligodactyly with thumb and 3 fingers on each hand was found in a Korean 77-year-old female donor. The anatomical structures were examined bilaterally in the hands and forearms of the individual. After documenting the characteristics of muscles, tendons, nerves, and arteries, they were all removed to leave only the bones. This process enabled a detailed analysis of the hand joints, the number and types of individual bones, as well as their shapes. This study received approval from the Institutional Review Board of the Sungkyunkwan University (IRB No. SKKU 2023-02-012; approval date: 8 February 2023).

## 3. Results

The donor exhibited bilateral hand oligodactyly, with each hand having a thumb and 3 fingers and both hands showing cleft-hand deformities, which were more severe on the right. Both forearms appeared normal.

### 3.1. Left Hand

#### 3.1.1. Forearm and Wrist

**Figure 1:**
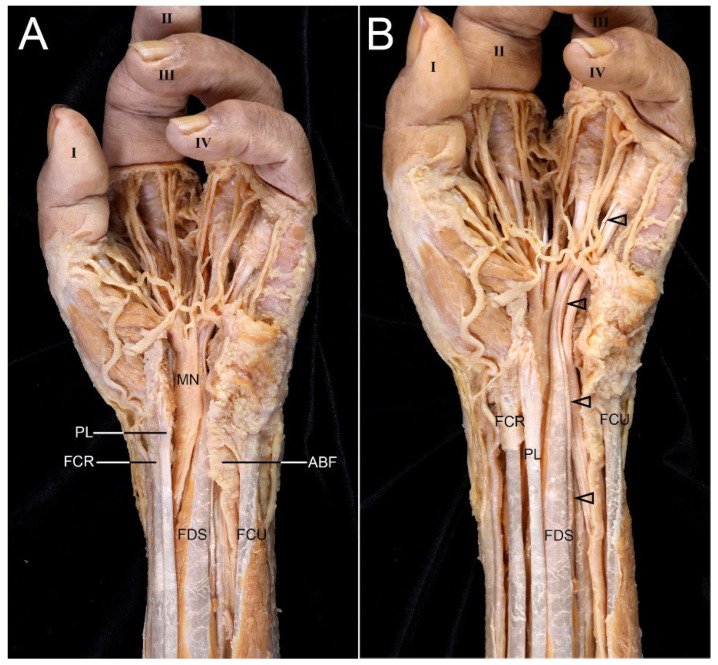
Anatomical findings for the forearm and wrist of the left hand with oligodactyly. The locations and shapes of all forearm muscles appeared normal. (**A**) The distal antebrachial fascia (ABF) was observed to be thickened. The median nerve (MN) was notably widened from this thickened fascia to the carpal tunnel. Cutting and removing the flexor retinaculum revealed the MN. (**B**) The tendon (arrowheads) of the flexor digitorum superficialis (FDS) that attached to the fourth digit was small and thin. FCR, flexor carpi radialis; PL, palmaris longus; FCU, flexor carpi ulnaris; I, first digit; II, second digit; III, third digit; IV, fourth digit.

The distal antebrachial fascia (ABF) was thickened, and the median nerve (MN) was widened toward the carpal tunnel (Figure 1A). Forearm muscles appeared normal, but the flexor digitorum superficialis (FDS) tendon for the fourth digit was thin (Figure 1B). The FDS tendons to the third and fourth digits were superficial, while that to the second was deeper. The pronator quadratus extended more proximally than usual.

#### 3.1.2. Palm

**Figure 2:**
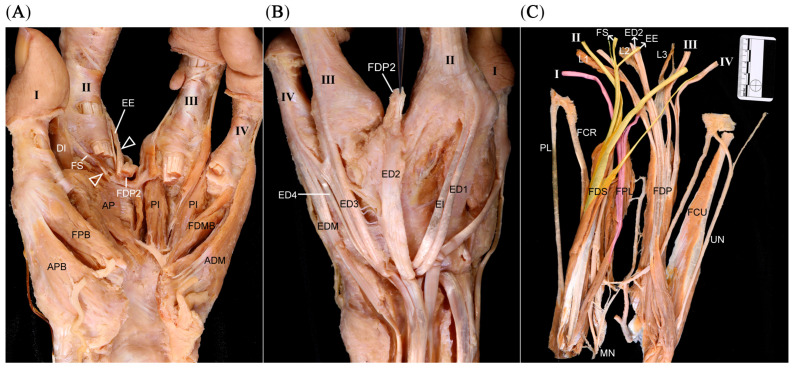
Anatomical findings for the palm of the left hand with oligodactyly. (**A**) Several tendinous slips (white arrowheads) of the FDS connecting to the second digit were observed. These slips were attached to the extensor expansion (EE) and the fibrous sheaths (FSs) of the metacarpophalangeal joint of the second digit. In the web space between the second and third digits, the second tendon of the flexor digitorum profundus (FDP2) extended dorsally. (**B**) FDP2 was attached to the extensor digitorum communis (ED) tendon that attached to the second digit (ED2, black arrowheads). (**C**) Removing the muscles and tendons of the digits and wrist revealed three lumbricals (L1, L2, and L3). The small arrows indicate the structures to which each tendinous slip and tendon connected. A connection (arrow) was observed between the tendons of the flexor pollicis longus (FPL) and the FDP. In panel C, the yellow color indicates the flexor digitorum superficialis (FDS) tendons, and the pink color indicates the flexor digitorum profundus (FDP) tendons. The colors are used solely to facilitate visual distinction. ADM, abductor digiti minimi; AP, adductor pollicis; APB, abductor pollicis brevis; DI, dorsal interosseous, ED1 and ED2, extensor digitorum tendons both attached to the second digit, ED3, extensor digitorum tendon attached to the third digit; ED4, extensor digitorum tendon attached to the fourth digit; EDM, extensor digiti minimi; FDMB, flexor digiti minimi; FPB, flexor pollicis brevis; PI, palmar interosseous; UN, ulnar nerve.

Several tendinous slips of the FDS attached to the extensor expansion (EE) and the metacarpophalangeal (MCP) joint of the second digit. In the web between the second and third digits, the second tendon of the FDP extended dorsally and attached to the extensor digitorum communis (ED) tendon that was attached to the second digit (Figure 2A,B). An unusual connection was observed between the FDP and the flexor pollicis longus (FPL) tendons (Figure 2C). Three lumbricals and three palmar interossei were identified.

#### 3.1.3. Dorsum

**Figure 3:**
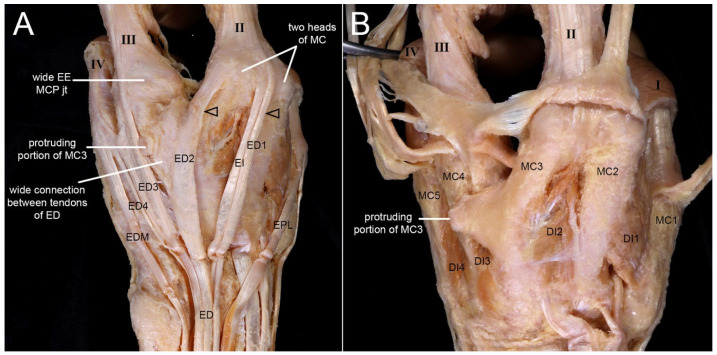
Metacarpal fusion and extensor tendon variations on the dorsum of the left hand with oligodactyly. (**A**) On the dorsum of the left hand, two metacarpals (second metacarpal [MC2] and third metacarpal [MC3]) joined at the MCP joint that was connected to the second digit. There were four ED tendons, but two tendons (arrowheads) inserted into the second digit. A protruding portion of MC3 was observed. (**B**) Reflecting the ED tendons on the dorsum allowed all five metacarpals to be identified. The heads of MC2 and MC3 were found to converge at the MCP joint of the second digit. A protruding portion of MC3 formed a transverse attachment with the fourth metacarpal (MC4). There were four dorsal interossei (DI) muscles. However, the third dorsal interosseous (DI3) muscle displayed thin and small muscle fibers, likely due to the protruding portion of MC3.

The MCP joint of the second digit was formed by two metacarpals (second metacarpal [MC2] and third metacarpal [MC3]), with dual ED tendons inserting on this digit (Figure 3A). Five metacarpals were confirmed, with a protruding third metacarpal [MC3] attached with the fourth metacarpal (MC4). Among four dorsal interossei, the third was small and thin (Figure 3B).

### 3.2. Right Hand

#### 3.2.1. Forearm and Wrist

Forearm muscles were typical. The ABF was thickened distally, but the MN appeared normal.

#### 3.2.2. Palm

**Figure 4:**
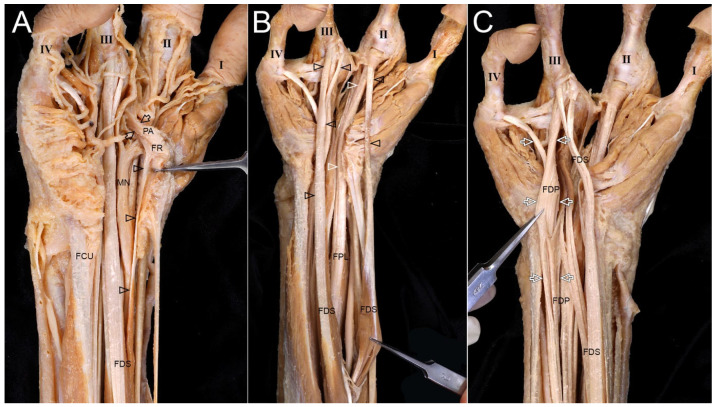
Tendinous slips and connections in the palm of the right hand with oligodactyly. (**A**) Cutting and reflecting the FR revealed a thin tendon (arrowheads) of the FDS attached to the deep surface of the FR. A portion of the palmar aponeurosis (PA, arrows) was attached to the deep structures, including the lumbrical between MC2 and MC3. (**B**) The FDS tendons (black arrowheads) attached to the second and third digits. The FPL tendon (white arrowheads) attached to the second digit, and there was no extrinsic tendon attached to the first digit. (**C**) The FDP tendons (white arrows) attached to the third and fourth digits. Both the FDS and FDP tendons were present for the third digit, while only the FDP tendon was observed attached to the fourth digit.

**Figure 5:**
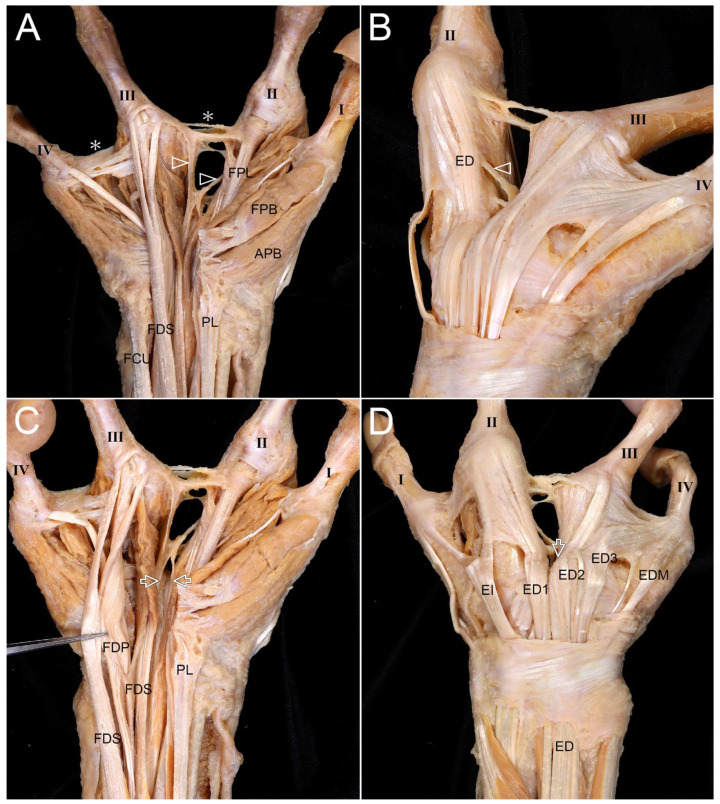
Flexor tendon variations in the palm of the right hand with oligodactyly. (**A**) Several tendinous slips (white arrowheads) arose from the FDP tendon between MC2 and MC3. Deep transverse metacarpal ligaments (asterisks) were distinctly prominent between the second and third digits and between the third and fourth digits. (**B**) Another slip (white arrowhead) was attached to the side of the ED tendon attached to the second digit. (**C**) A tendon of the FDP (arrows) between MC2 and MC3 extended distally and entered the dorsum of the hand. (**D**) This portion of the FDP tendon (arrow) was then attached to ED2 for the third digit.

**Figure 6:**
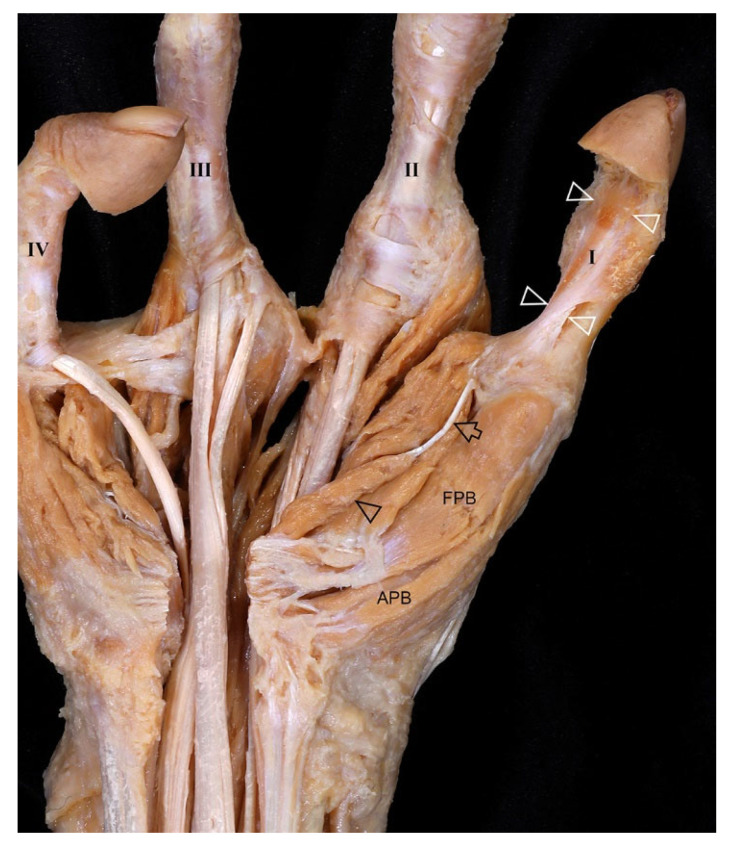
First-digit tendon variations in the palm of the right hand with oligodactyly. A tendon (white arrowheads) attached to the first digit originated at the MCP joint and attached to the distal phalanx, while the FPL tendon extended to the second digit. In addition, a thin and slender muscle bundle (black arrowhead) arose from the FR, forming a long and delicate tendon (arrow) that attached to the MCP joint of the first digit.

A thin FDS tendon was attached to the deep side of the flexor retinaculum (FR), and the palmar aponeurosis extended into deep structures (Figure 4A). FDS tendons inserted into the second and third digits, with one dividing into two slips at the third digit (Figure 4B). The FPL tendon inserted into the second digit, while no extrinsic tendon attached to the first digit. FDP tendons attached to the third and fourth digits (Figure 4C). Multiple tendinous slips from the FDP extended toward the ED, thick fascia that covered the surface of MC3, and deep transverse metacarpal ligament of digit (Figure 5). A delicate tendon from the FR inserted into the MCP joint of the first digit (Figure 6).

**Figure 7:**
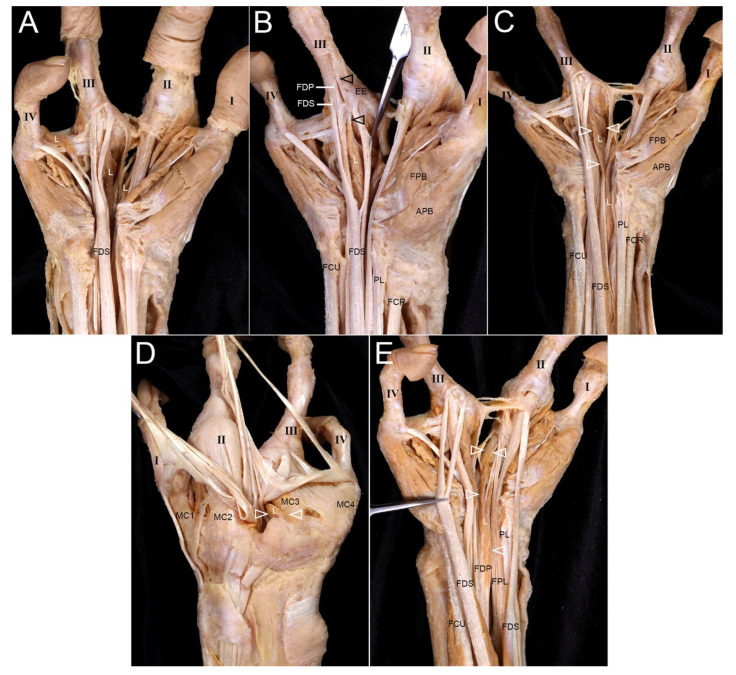
Variations in L (lumbrical muscles) in the right hand with oligodactyly. (**A**) The right hand contained three lumbrical muscles. (**B**) One lumbrical originated from the combined tendon of the FDS and FDP in the distal forearm. The lumbrical tendon (arrowheads) formed part of the flexor tendon attached to the third digit and also attached to the EE. (**C**) The lumbrical (arrowheads) was in a lambda shape, with a portion extending to the dorsum of the hand. (**D**) On the dorsum, part of the lumbrical (arrowheads) attached to the base of MC3 in an abnormal shape. (**E**) Another lumbrical (arrowheads) arose from the FDP tendon proximal to the wrist joint.

Three lumbricals were observed. One formed a lambda shape, extending to the dorsum and EE of the third digit. Another arose proximally and attached to fascia over MC2 (Figure 7). Deep transverse metacarpal ligaments were prominent.

#### 3.2.3. Dorsum

**Figure 8:**
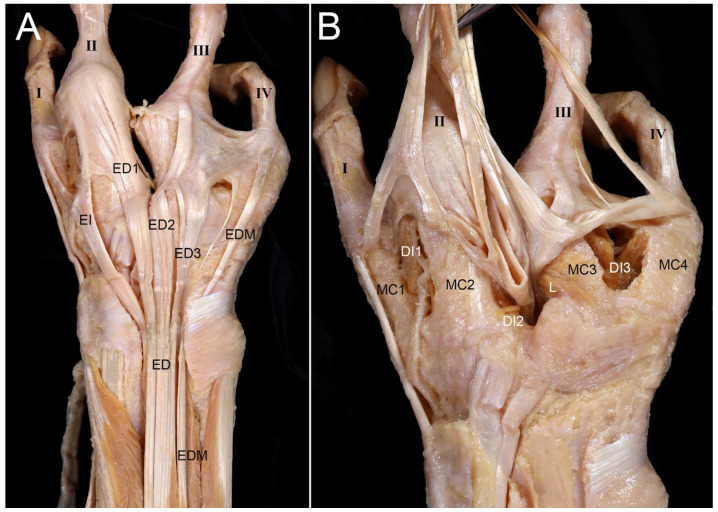
Anatomical findings for the dorsum of the right hand with oligodactyly. (**A**) On the dorsum of the hand, the extensor indicis proprius (EI) tendon was divided into two slips that attached to both the first and second digits. There were three ED tendons, with the ED3 tendon for the 4th digit following a curved course. (**B**) Four metacarpals and three DI muscles were present. The web space between the second and third digits was larger than normal, extending as far as the carpometacarpal joint and becoming deeper. The fascia on the dorsal and side surfaces of the metacarpal bones was distinct and thickened, resembling the EE. The tendons on the dorsum of the hand were retracted distally to reveal the metacarpals and DI muscles. DI1, first dorsal interosseous; DI2, second dorsal interosseous; DI3, third dorsal interosseous; L, lumbrical.

The EI tendon divided, attaching to the first and second digits. Three ED tendons were present, with one coursing obliquely to the fourth digit. A cleft extended deeply between the second and third digits (Figure 8).

### 3.3. Bones of the Left and Right Hands

**Figure 9:**
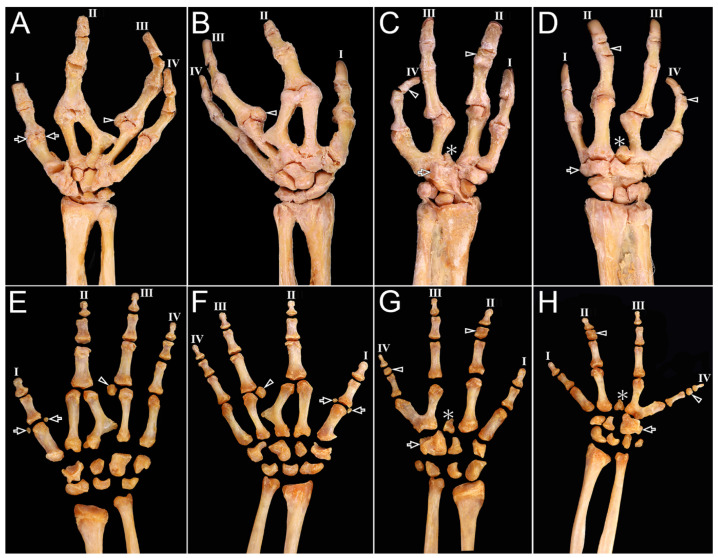
Bones of the left and right hands with oligodactyly. Ventral and dorsal views of the articulations of the left hand (**A**,**B**) and right hand (**C**,**D**), and of the separated bones of the left hand (**E**,**F**) and right hand (**G**,**H**). The left hand had five metacarpal bones. MC2 and MC3 joined at the MCP joint, and the third digit had a small bone (arrowhead) at its MCP joint. Two sesamoid bones (arrows) were present at the MCP joint of the first digit. The proximal phalanx of the second digit, which articulated with two metacarpals, was found to be hypertrophied. The carpal bones included seven bones, with the pisiform absent and the presence of variations in shape and size (**A**,**B**,**E**,**F**). The right hand had four metacarpal bones, with the bases of MC3 and MC4 fused, forming a partial synostosis. A small bone (asterisks) was present between the bases of the metacarpals connected to the second and third digits and the carpal bones, which appeared to be aplasia of the metacarpal shaft. The middle phalanges (arrowheads) of the second and fourth digits were shorter, and did not form the typical shape of long bones. The seven carpal bones of the wrist exhibited atypical morphology, including capitohamate fusion. Unlike in the left hand, the pisiform bone was present and MC2 had a larger-than-normal base (**C**,**D**,**G**,**H**).

The left hand contained five metacarpals with a horizontal Y-shaped configuration of MC3 and hypertrophy of the second proximal phalanx. The right hand had four metacarpals with a V-shaped fusion between MC3–MC4 and a small accessory bone between MC2–MC3 and the carpus. Capitohamate fusion was present on the right; pisiform was absent on the left (Figure 9).

## 4. Discussion

**Figure 10:**
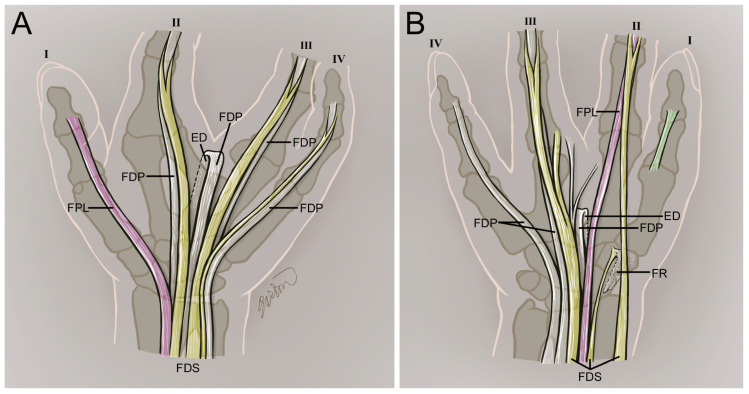
Schematic summarizing the key tendon and skeletal variations observed in bilateral hand oligodactyly. (**A**) Left hand demonstrating variations in the FDS, FDP, and FPL tendons, along with metacarpal and phalangeal anomalies. (**B**) Right hand illustrating altered tendon attachments and metacarpal–carpal variations. FDS tendons are shown in yellow, FDP tendons in white, and the FPL tendon in pink. The tendon supplying the first digit on the right hand is shown in green.

This study investigated the rare case of bilateral hand oligodactyly, revealing asymmetrical malformations in bones, muscles, and tendons. Structural anomalies differed significantly between hands. These findings, summarized in Table 1 and Figure 10, provide insights into the variability and developmental complexity of this congenital condition.

In the left hand, MC2 and MC3 articulated with the proximal phalanx of the second digits. The right hand also displayed significant abnormalities, with the absence of one metacarpal resulting in only four metacarpals. This deficiency appeared to have contributed to the cleft-hand deformity being more pronounced in the right hand. An additional small bone was observed in the right hand between the bases of MC2 and MC3 and the carpal bones, which might have arisen from incomplete development of the metacarpals.

Grasping and pinching are fundamental functions of the hand [17], which might have been compromised in the present case. Initiating grasp movements requires finger extension through contraction of the intrinsic hand muscles with flexion of the MCP joint [18,19]. In the present case, the abnormal attachment of the lumbricals might have hindered the proper initiation of the grasp movement. The abnormal connection between the FDP and the extensor might also have disrupted force transmission so as to reduce the grip strength. Furthermore, the absence of one or more fingers in hand oligodactyly leads to a decrease in flexion strength [20]. The pinch movement occurs when the first digit opposes and contacts the second and third digits to grasp an object [21]. The donor in the present study had a second digit that showed radial deviation and a smaller first web space, suggesting that pinch movements would have been performed using the third and fourth digits. Thickening of the second digit might also have hindered pinch movements, potentially affecting actions such as holding a pen [22].

Baek and Kim (2016) [5] classified oligodactyly with a first digit into two types based on the morphology of the thumb and the pattern of missing digits: Type I, featuring a normal first digit, and Type II, characterized by first-digit hypoplasia. In the present donor, the left hand corresponded to Type I, with a normal first digit and an absent central digit, whereas the right hand corresponded to Type II, showing first-digit hypoplasia and a more-severe central ray deficiency. The metacarpals were hypertrophied in cases with a missing central digit in a previous report [5], and the metacarpals in the present donor showed abnormal configurations: horizontal Y and V shapes between the MC2 and MC3 and between the MC3 and MC4 in the left and right hands, respectively. A hypertrophied proximal phalanx of the second digit of the left hand, which articulated with two metacarpals, was observed in the current study.

Conrad and Ezaki (2002) [1] demonstrated a spectrum of oligodactyly in cleft hands, ranging from central clefts with digit incorporation to severe digit loss. In the present donor, the left and right hands exhibited central clefts extending to the metacarpal bases, with four digits remaining, aligning with the type of cleft hand with four digits of the previous report [1]. Swanson et al. (1984) [14] found that capitohamate fusion predominated in the cases of total or partial defects of the ulna with humeroradial synostosis, though trapezium–scaphoid fusion was observed in some instances. Havenhill et al. (2005) [8] similarly reported the capitohamate fusion in a case of ulnar longitudinal deficiency. While the current case involved central ray deficiency rather than ulnar longitudinal deficiency, capitohamate fusion was observed in the right hand.

Tada (1984) [15] reported operative findings for the cases of central ray deficiency of the hand, distinguishing between deficiency and syndactylous types. In the deficiency type, the long flexor and extensor tendons were inserted directly at the bony-defect stump, without connections to adjacent tendons. In contrast, the syndactylous type displayed partial insertion of the flexor and extensor tendons at the bony stump of the third digit, with additional tendinous connections to the adjacent fourth digit. In both the left and right hands of the present case, the FDP tendon crossed dorsally to connect to the ED tendon, which represents an unusual anatomical variation. Additionally, in the left hand, a tendinous slip of the FDS tendon to the second digit was observed attaching to the MCP joint of the same digit. In the right hand, the EI tendon divided into two slips that attached to the first and second digits. The FPL tendon typically inserts into the first digit, but in the right hand this was instead redirected to the second digit, likely resulting in impairments of first-digit flexion, grip strength, and fine motor control.

Limb development and digit development are orchestrated by key signaling centers that coordinate growth along three axes: proximodistal (shoulder to fingertip), anteroposterior (first to fifth digit), and dorsoventral (dorsum of the hand to palm). The apical ectodermal ridge (AER) secretes fibroblast growth factors (FGFs) that promote limb elongation along the proximodistal axis, while the ZPA (zone of polarizing activity) releases Sonic Hedgehog (Shh) that guides anteroposterior patterning [23,24]. The dorsoventral polarity is established by Wnt7a and EN1 signals in the ectoderm [25]. These pathways interact via feedback loops, such as Shh inducing FGF4 in the AER, ensuring coordinated limb development [26]. Disruptions to these mechanisms can lead to defects such as polydactyly (extra digits) or syndactyly (fused digits) [27]. In addition, Wnt signaling, HOX genes, and BMP (bone morphogenetic protein) signaling are associated with congenital limb malformations [28]. Specifically, hand oligodactyly is considered to result from defects in SHH-FGF signaling—which plays crucial roles in cell proliferation and differentiation—that lead to incomplete hand development [29,30].

In conclusion, hand oligodactyly is not merely the incomplete development of bone, but also includes malformation of all related structures including muscles and tendons, as demonstrated in this case of cleft hand. The results of this study have provided valuable anatomical insights into this malformation and useful reference information for understanding its structural complexity, and are expected to help design appropriate surgical treatments.

## Figures and Tables

**Table 1:** Structural variations in bilateral hand oligodactyly.

	Left	Right
Palmar Aponeurosis	Normal	Attached to the Deep Structures Between MC2 and MC3
Wrist	ABF thicker at the distal forearm	
Median nerve	Wider at the carpal tunnel and distal forearm	Normal
Radial artery	A distinct branch of the radial artery coursed on the thenar muscles	Normal
Forearm muscles	Normal	Normal
FDS tendon	4	3
FDP tendon	4 (attached to the ED tendon)	3 (attached to the ED tendon)
FPL	Normal	connected to the second digit (like an FDP tendon)
Intertendinous connection between FPL and FDP	Present	Normally absent
PQ	Extended proximally	Normal
PI	3	3
Lumbrical	3	3 (abnormal shapes and attachments)
Deep transverse metacarpal ligament	Distinct and thick between the second and third digits and between the third and fourth digits	
ED	4	3
Extensor indicis proprius	Normal	First and second digits
Metacarpal	5	4
DI muscles	4 (first DI had a hypertrophied portion that attached to the radial side of the second digit)	3 (smaller)
Tendon attached to the first digit	Normal	From MCP junction to distal phalanx
Carpal bone	7 (pisiform absent)	7 (capitohamate fusion)
Other	Complicated structures between MC2 and MC3	

ABF, antebrachial fascia; FDS, flexor digitorum superficialis; FDP, flexor digitorum profundus; MCP, metacarpophalangeal; FPL, flexor pollicis longus; ED, extensor digitorum communis; DI, dorsal interosseous; MC2, second metacarpal; MC3, third metacarpal; PQ, pronator quadratus; PI, palmar interosseous. Each number indicates the number of each structure.

## Data Availability

Data are available at the authors by reasonable request.
